# Supplementary material for: Performance of fecal inflammatory biomarkers to identify watery shigellosis: Findings from the Enterics for Global Health (EFGH) Shigella surveillance study
Source: PLoS Negl Trop Dis. 2026 Jun 1;20(6):e0014025. doi: 10.1371/journal.pntd.0014025 (PMC13235866; doi:10.1371/journal.pntd.0014025)
Supplement: S1 Appendix — Fig B. Diagnostic characteristics of the biomarkers individually to identify watery bacterial diarrhea by qPCR. Fig C. Variable importance in the prediction of watery bacterial diarrhea. Fig D. ROC curves for watery bacterial diarrhea prediction models with and without fecal biomarkers. Table A. Model Performance in the prediction of watery diarrhea attributed to Shigella when constraining specificity to 0.8. Table B. Model performance in the prediction of Shigella attributable diarrhea by culture. Table C. Model Performance in the prediction of watery diarrhea attributed to Shigella when excluding Shigella transmission season indicator. Table D. Model performance in the prediction of Shigella attributable diarrhea by qPCR stratified by age. Table E. Model performance in the prediction of Shigella attributable diarrhea by qPCR stratified by symptom duration. Table F. Comparison of bootstrapped sensitivities and specificities for watery shigellosis prediction with iterative biomarker inclusion. Table G. Model Performance in the prediction of watery bacterial diarrhea by qPCR. Table H. Comparison of bootstrapped AUCs for watery bacterial diarrhea prediction with iterative biomarker inclusion. Table I. Comparison of bootstrapped sensitivities and specificities for watery bacterial diarrhea prediction with iterative biomarker inclusion. (DOCX) [file pntd.0014025.s001.docx]

## Supplemental Material


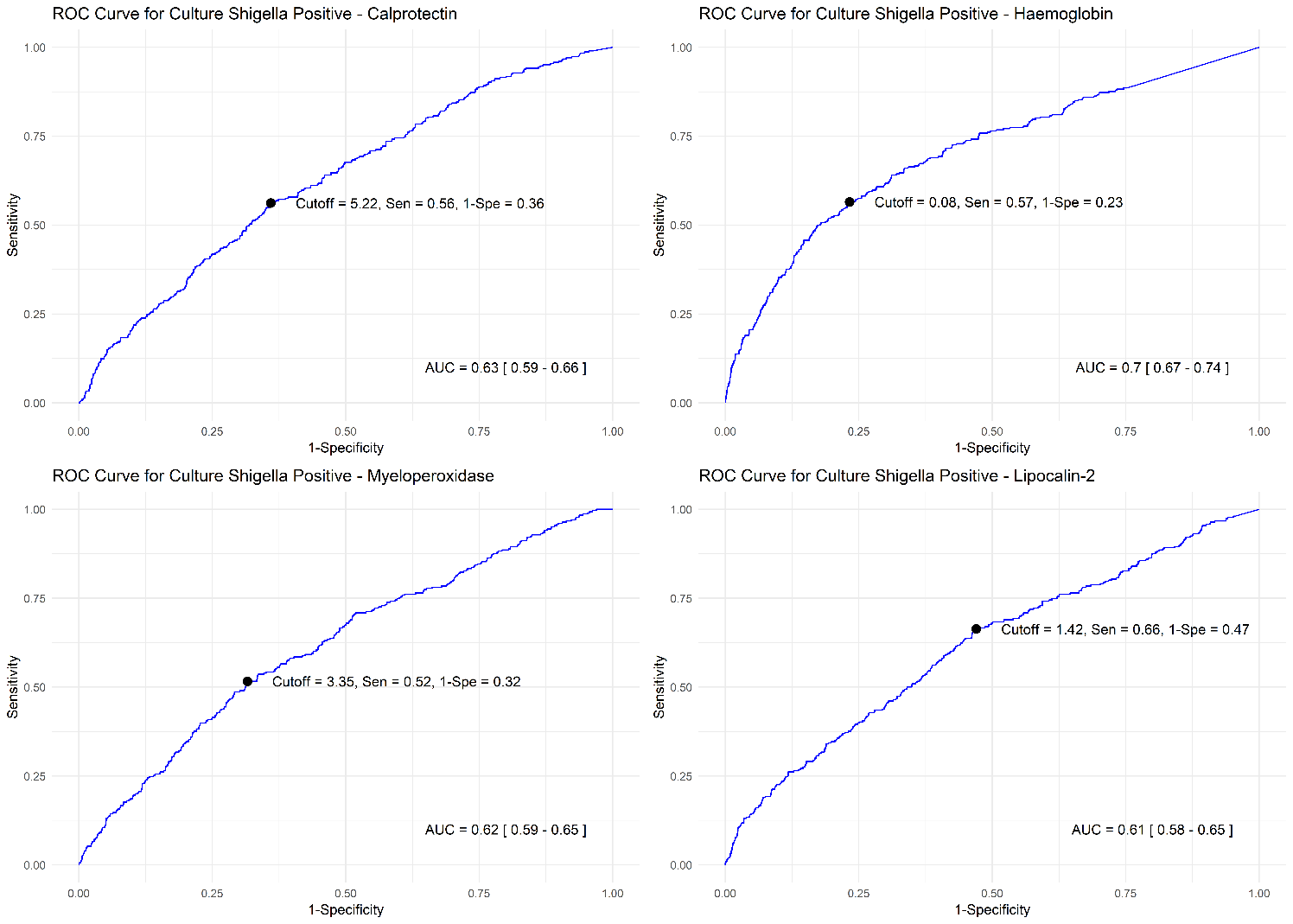


Fig A. Diagnostic characteristics of the biomarkers individually to identify watery diarrhea attributed to *Shigella* by culture


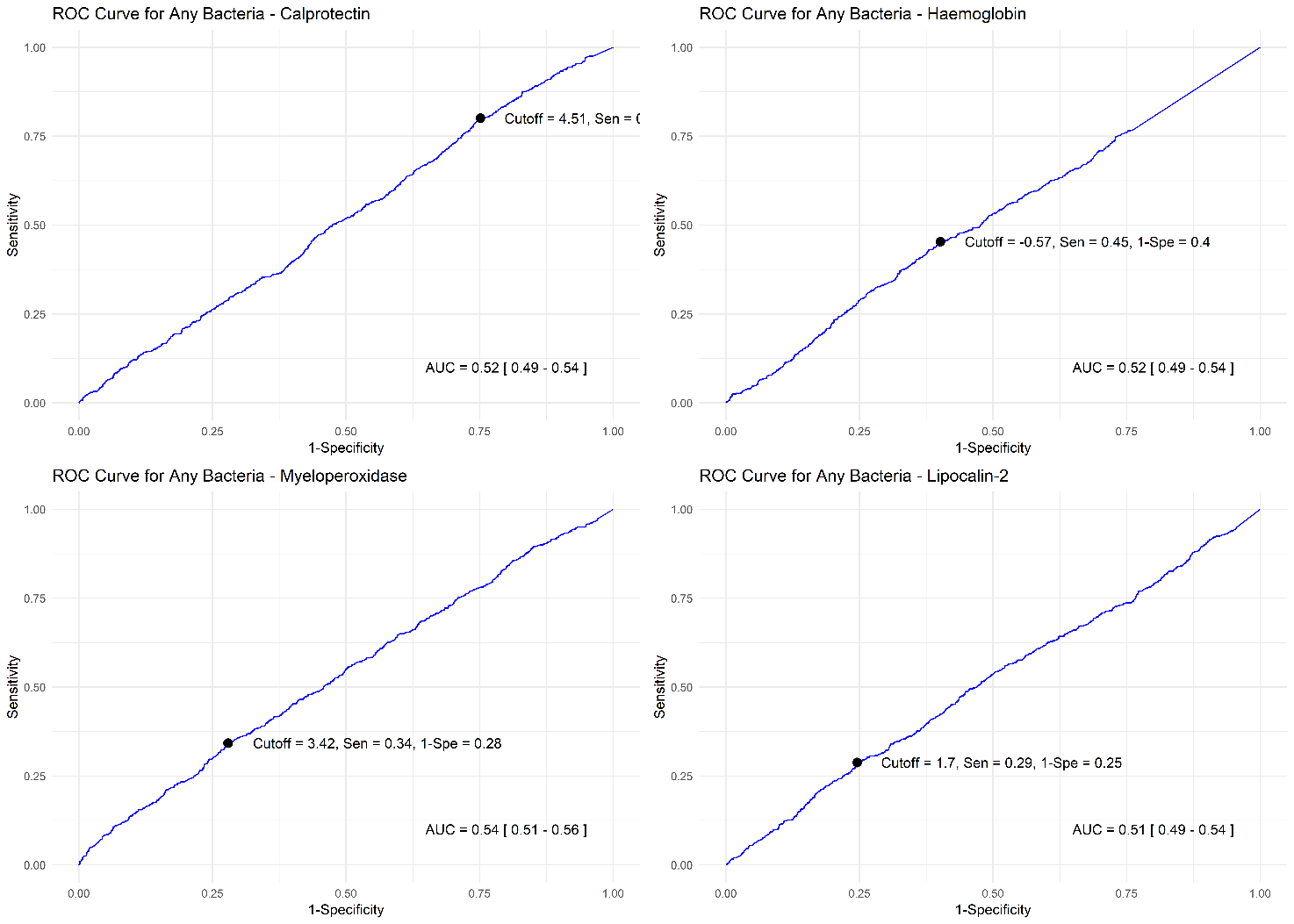


Fig B. Diagnostic characteristics of the biomarkers individually to identify watery bacterial diarrhea by qPCR


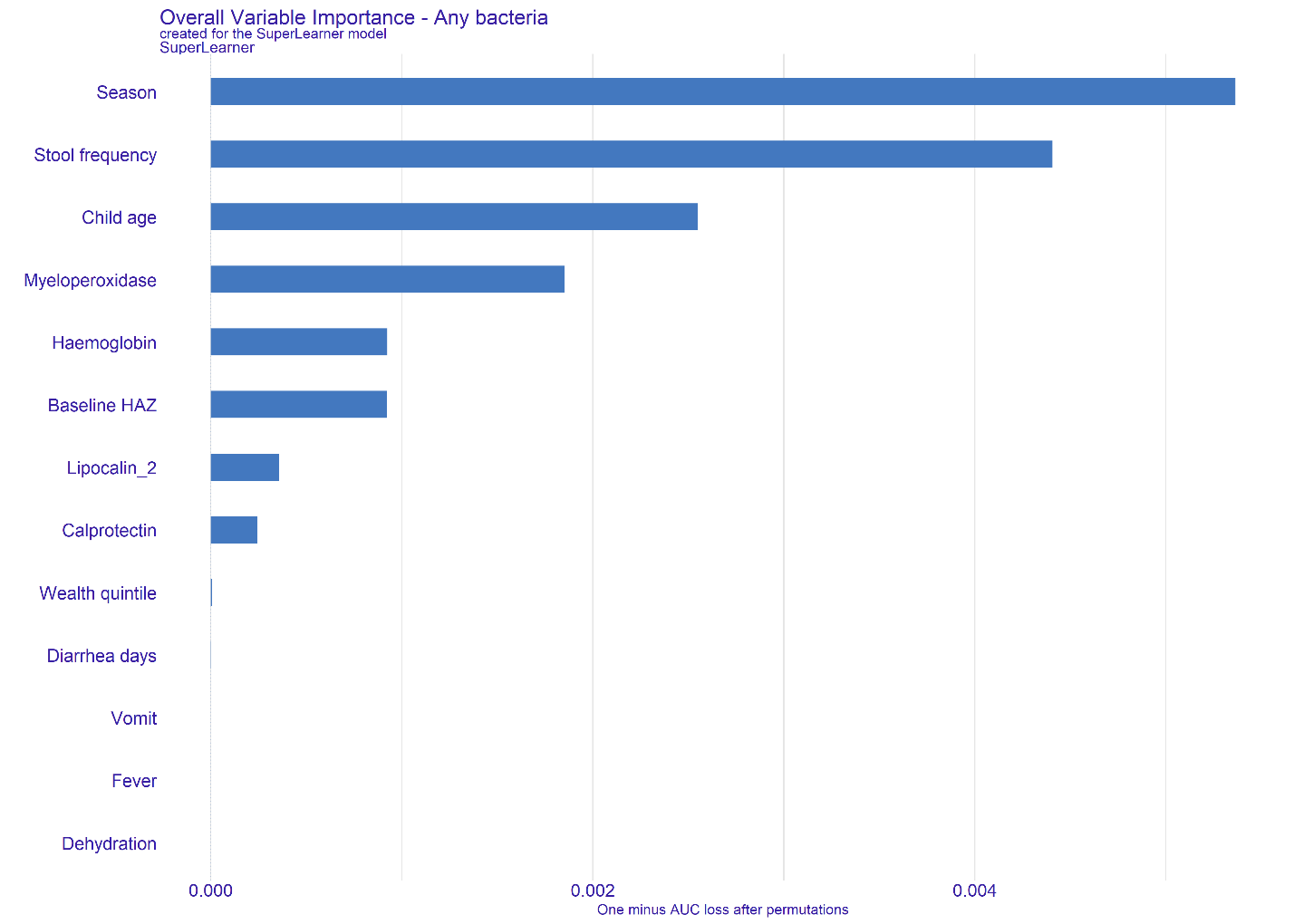


Fig C. Variable importance in the prediction of watery bacterial diarrhea


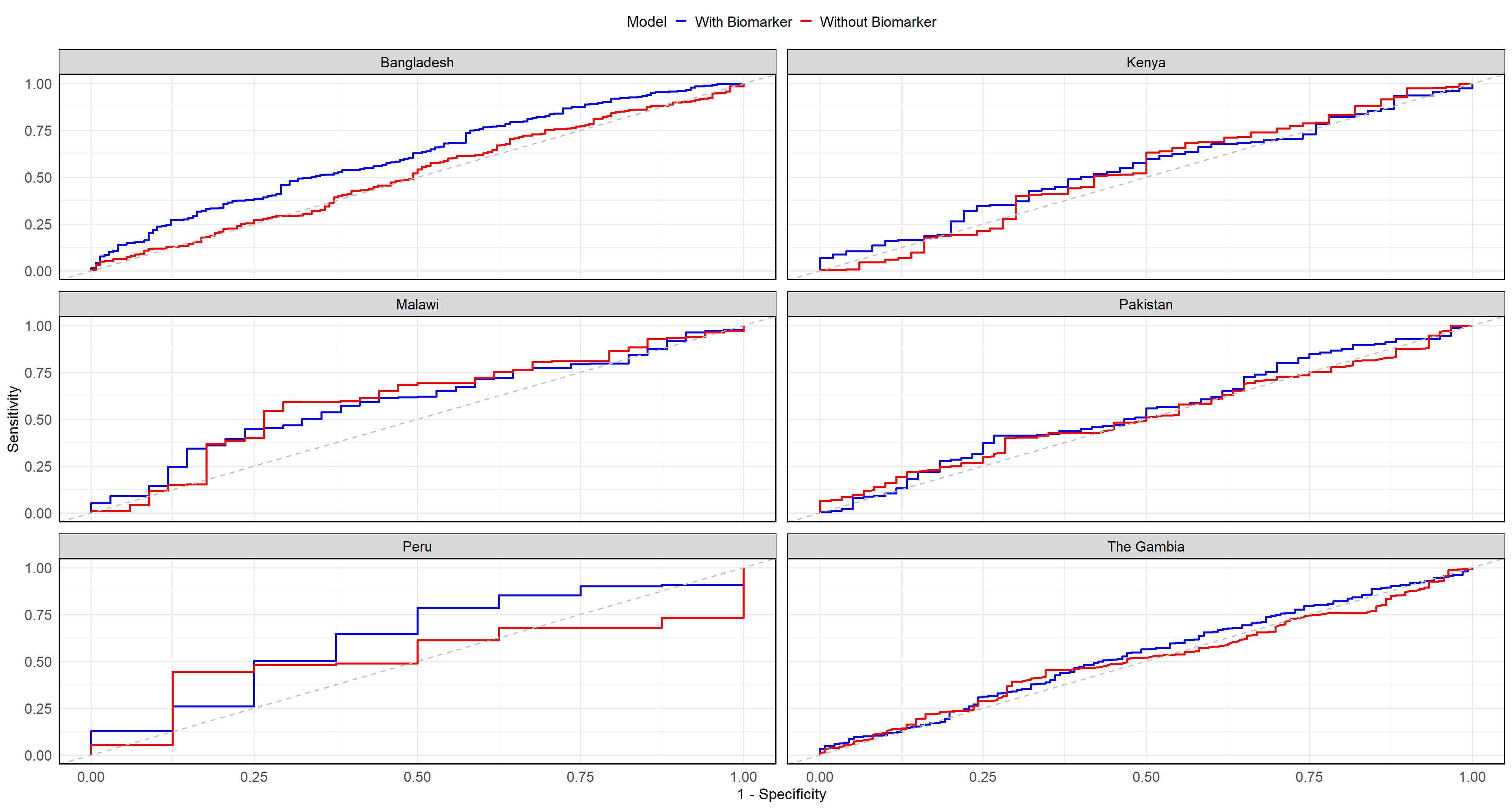


Fig D. ROC curves for bacterial diarrhea prediction models with and without fecal biomarkers

Table A. Model Performance in the prediction of watery diarrhea attributed to *Shigella* when constraining specificity to 0.8

| **Including biomarkers and clinical and socio-demographic predictors** | | | | | |
| --- | --- | --- | --- | --- | --- |
| **Validation site** | **Sensitivity [95% CI]** | **Specificity [95% CI]** | **PPV [95% CI]** | **NPV [95% CI]** | **AUC [95% CI]** |
| Bangladesh | 0.54 [0.48-0.60] | 0.80 [0.80-0.81] | 0.43 [0.40-0.47] | 0.86 [0.84-0.88] | 0.72 [0.69-0.74] |
| Kenya | 0.61 [0.53-0.70] | 0.81 [0.80-0.82] | 0.31 [0.26-0.35] | 0.94 [0.92-0.95] | 0.78 [0.73-0.82] |
| Malawi | 0.64 [0.53-0.74] | 0.81 [0.80-0.84] | 0.24 [0.19-0.29] | 0.96 [0.95-0.97] | 0.77 [0.71-0.83] |
| Pakistan | 0.57 [0.47-0.66] | 0.81 [0.80-0.82] | 0.45 [0.40-0.51] | 0.87 [0.84-0.90] | 0.74 [0.69-0.78] |
| Peru | 0.35 [0.26-0.45] | 0.81 [0.80-0.85] | 0.28 [0.21-0.34] | 0.86 [0.84-0.90] | 0.66 [0.61-0.72] |
| The Gambia | 0.56 [0.50-0.63] | 0.80 [0.80-0.81] | 0.46 [0.42-0.50] | 0.86 [0.84-0.88] | 0.76 [0.73-0.79] |
| Overall | 0.56 [0.38-0.63] | 0.81 [0.80-0.82] | 0.37 [0.25-0.46] | 0.87 [0.86-0.96] | 0.75 [0.67-0.78] |
| **Excluding biomarkers** | | | | | |
| **Validation site** | **Sensitivity [95% CI]** | **Specificity [95% CI]** | **PPV [95% CI]** | **NPV [95% CI]** | **AUC [95% CI]** |
| Bangladesh | 0.45 [0.39-0.51] | 0.80 [0.80-0.81] | 0.39 [0.35-0.43] | 0.84 [0.82-0.86] | 0.68 [0.65-0.71] |
| Kenya | 0.34 [0.26-0.42] | 0.81 [0.80-0.83] | 0.20 [0.15-0.24] | 0.90 [0.88-0.92] | 0.68 [0.63-0.72] |
| Malawi | 0.40 [0.29-0.51] | 0.81 [0.80-0.84] | 0.17 [0.12-0.22] | 0.94 [0.92-0.95] | 0.67 [0.62-0.73] |
| Pakistan | 0.36 [0.29-0.44] | 0.81 [0.80-0.82] | 0.35 [0.29-0.40] | 0.82 [0.79-0.85] | 0.63 [0.59-0.68] |
| Peru | 0.28 [0.17-0.36] | 0.81 [0.80-0.82] | 0.22 [0.14-0.28] | 0.85 [0.82-0.88] | 0.61 [0.56-0.66] |
| The Gambia | 0.47 [0.41-0.53] | 0.80 [0.80-0.82] | 0.42 [0.37-0.46] | 0.83 [0.81-0.86] | 0.71 [0.67-0.74] |
| Overall | 0.38 [0.28-0.47] | 0.81 [0.80-0.81] | 0.28 [0.17-0.42] | 0.84 [0.82-0.93] | 0.67 [0.61-0.70] |

PPV- Positive Predictive value; NPV-Negative Predictive value; AUC- Area under the Curve

Table B. Model performance in the prediction of *Shigella* attributable diarrhea by culture

| **Cutoff** | **Sensitivity [95% CI]** | **Specificity [95% CI]** | **PPV [95% CI]** | **NPV [95% CI]** | **AUC [95% CI]** |
| --- | --- | --- | --- | --- | --- |
| Youden's Index | 0.69 [0.58- 0.81] | 0.85 [0.73- 0.88] | 0.25 [0.16- 0.30] | 0.97 [0.96- 0.98] | 0.79 [0.74- 0.85] |
| Constraining  Specificity to 0.8 | 0.69 [0.59- 0.79] | 0.81 [0.80- 0.84] | 0.21 [0.17- 0.26] | 0.97 [0.96- 0.98] | 0.79 [0.74- 0.85] |

PPV- Positive Predictive value; NPV-Negative Predictive value; AUC- Area under the Curve

Table C. Model Performance in the prediction of watery diarrhea attributed to *Shigella* when *excluding Shigella* transmission season indicator

| **Youden's Index** | | | | | |
| --- | --- | --- | --- | --- | --- |
| **Validation site** | **Sensitivity [95% CI]** | **Specificity [95% CI]** | **PPV [95% CI]** | **NPV [95% CI]** | **AUC [95% CI]** |
| Bangladesh | 0.60 [0.47-0.84] | 0.74 [0.48-0.85] | 0.38 [0.30-0.46] | 0.87 [0.84-0.92] | 0.71 [0.68-0.74] |
| Kenya | 0.74 [0.63-0.83] | 0.76 [0.69-0.84] | 0.30 [0.24-0.38] | 0.95 [0.94-0.97] | 0.78 [0.73-0.82] |
| Malawi | 0.59 [0.47-0.84] | 0.88 [0.62-0.93] | 0.31 [0.15-0.43] | 0.96 [0.94-0.98] | 0.76 [0.70-0.82] |
| Pakistan | 0.62 [0.47-0.74] | 0.73 [0.61-0.85] | 0.40 [0.33-0.51] | 0.87 [0.84-0.90] | 0.71 [0.66-0.75] |
| Peru | 0.42 [0.27-0.87] | 0.81 [0.11-0.93] | 0.30 [0.14-0.43] | 0.88 [0.77-0.94] | 0.60 [0.46-0.66] |
| The Gambia | 0.57 [0.48-0.78] | 0.79 [0.57-0.85] | 0.45 [0.34-0.52] | 0.86 [0.84-0.90] | 0.72 [0.69-0.76] |
| Overall | 0.61 [0.50-0.72] | 0.75 [0.69-0.84] | 0.34 [0.29-0.44] | 0.88 [0.86-0.96] | 0.72 [0.60-0.78] |
| **Constraining specificity to 0.8** | | | | | |
| **Validation site** | **Sensitivity [95% CI]** | **Specificity [95% CI]** | **PPV [95% CI]** | **NPV [95% CI]** | **AUC [95% CI]** |
| Bangladesh | 0.49 [0.44-0.55] | 0.80 [0.80-0.81] | 0.41 [0.38-0.45] | 0.85 [0.83-0.87] | 0.71 [0.68-0.74] |
| Kenya | 0.67 [0.58-0.74] | 0.81 [0.80-0.82] | 0.33 [0.28-0.37] | 0.94 [0.93-0.96] | 0.78 [0.73-0.82] |
| Malawi | 0.60 [0.49-0.70] | 0.82 [0.81-0.85] | 0.24 [0.19-0.30] | 0.96 [0.94-0.97] | 0.76 [0.70-0.82] |
| Pakistan | 0.51 [0.43-0.59] | 0.81 [0.80-0.82] | 0.43 [0.37-0.48] | 0.85 [0.83-0.88] | 0.71 [0.66-0.75] |
| Peru | 0.38 [0.18-0.48] | 0.82 [0.80-0.92] | 0.29 [0.21-0.35] | 0.87 [0.84-0.90] | 0.60 [0.46-0.66] |
| The Gambia | 0.54 [0.48-0.60] | 0.80 [0.80-0.81] | 0.45 [0.41-0.49] | 0.85 [0.83-0.88] | 0.72 [0.69-0.76] |
| Overall | 0.52 [0.38-0.65] | 0.81 [0.80-0.82] | 0.37 [0.25-0.45] | 0.86 [0.85-0.95] | 0.72 [0.60-0.78] |

Table D. Model performance in the prediction of *Shigella* attributable diarrhea by qPCR stratified by age

| **Cutoff** | **Age** | **Sensitivity [95% CI]** | **Specificity [95% CI]** | **PPV [95% CI]** | **NPV [95% CI]** | **AUC [95% CI]** |
| --- | --- | --- | --- | --- | --- | --- |
| Youden | 6-11m | 0.92 [0.83- 1.00] | 0.60 [0.56- 0.67] | 0.16 [0.11- 0.20] | 0.99 [0.98- 1.00] | 0.72 [0.65- 0.77] |
|  | 12-23m | 0.85 [0.62- 0.96] | 0.58 [0.43- 0.80] | 0.34 [0.28- 0.45] | 0.94 [0.89- 0.98] | 0.78 [0.74- 0.82] |
|  | 24-35m | 0.85 [0.50- 1.00] | 0.62 [0.51- 0.93] | 0.46 [0.37- 0.72] | 0.92 [0.83- 1.00] | 0.78 [0.71- 0.85] |
| Constraining  Specificity to 0.8 | 6-11m | 0.27 [0.11- 0.47] | 0.81 [0.80- 0.87] | 0.10 [0.05- 0.16] | 0.93 [0.91- 0.96] | 0.72 [0.65- 0.77] |
|  | 12-23m | 0.55 [0.44- 0.64] | 0.81 [0.80- 0.82] | 0.42 [0.35- 0.48] | 0.88 [0.84- 0.91] | 0.78 [0.74- 0.82] |
|  | 24-35m | 0.52 [0.39- 0.67] | 0.83 [0.80- 0.87] | 0.53 [0.42- 0.63] | 0.82 [0.77- 0.88] | 0.78 [0.71- 0.85] |

PPV- Positive Predictive value; NPV-Negative Predictive value; AUC- Area under the Curve

Table E. Model performance in the prediction of *Shigella* attributable diarrhea by qPCR stratified by symptom duration

| Cutoff | Age | Sensitivity [95% CI] | Specificity [95% CI] | PPV [95% CI] | NPV [95% CI] | AUC [95% CI] |
| --- | --- | --- | --- | --- | --- | --- |
| Youden | ≤2 days | 0.83 [0.66-0.95] | 0.65 [0.55-0.80] | 0.28 [0.21-0.37] | 0.96 [0.93-0.99] | 0.79 [0.73-0.84] |
|  | ≥3 days | 0.69 [0.62-0.78] | 0.79 [0.71-0.84] | 0.43 [0.35-0.50] | 0.92 [0.90-0.94] | 0.77 [0.73-0.81] |
| Constraining Specificity to 0.8 | ≤2 days | 0.56 [0.38-0.71] | 0.81 [0.80-0.84] | 0.32 [0.24-0.39] | 0.92 [0.89-0.95] | 0.79 [0.73-0.84] |
|  | ≥3 days | 0.66 [0.58-0.74] | 0.81 [0.80-0.82] | 0.44 [0.39-0.49] | 0.91 [0.89-0.93] | 0.77 [0.73-0.81] |

Table F. Comparison of bootstrapped sensitivities and specificities for watery shigellosis prediction with iterative biomarker inclusion

| **Metric** | **Validation site** | **Incremental mean difference in AUC (95% CI)** | | | |
| --- | --- | --- | --- | --- | --- |
|  |  | **Clinical predictors + HAEM vs. Clinical predictors** | **Clinical predictors + HAEM**  **and MPO vs. Clinical predictors + HAEM** | **Clinical predictors + HAEM, MPO, CAL vs. Clinical predictors + HAEM + MPO** | **Clinical predictors + HAEM, MPO, CAL, NGAL vs. Clinical predictors + HAEM + MPO + CAL** |
| Sensitivity | Bangladesh | 0.11 [0.06-0.17] | 0.09 [0.06-0.13] | -0.10 [-0.14--0.06] | 0.16 [0.12-0.19] |
|  | Kenya | -0.15 [-0.23--0.07] | 0.30 [0.22-0.37] | -0.12 [-0.17--0.07] | -0.04 [-0.09-0.00] |
|  | Malawi | 0.04 [-0.04-0.12] | -0.18 [-0.26--0.09] | -0.04 [-0.1-0.03] | -0.04 [-0.07-0.00] |
|  | Pakistan | 0.17 [0.10-0.23] | 0.04 [-0.02-0.1] | -0.01 [-0.04-0.03] | -0.02 [-0.07-0.03] |
|  | Peru | 0.03 [-0.05-0.11] | 0.06 [0.02-0.11] | -0.03 [-0.06-0.00] | 0.02 [-0.03-0.05] |
|  | The Gambia | -0.03 [-0.08-0.03] | 0.05 [0.01-0.1] | 0.13 [0.09-0.17] | -0.03 [-0.05-0.00] |
|  | Overall | -0.03 [-0.08-0.03] | 0.05 [0.01-0.1] | 0.13 [0.09-0.17] | -0.03 [-0.05-0.00] |
| Specificity | Bangladesh | -0.03 [-0.05-0.00] | -0.11 [-0.13--0.09] | 0.11 [0.09-0.13] | -0.12 [-0.14--0.1] |
|  | Kenya | 0.22 [0.19-0.24] | -0.23 [-0.25--0.2] | 0.07 [0.05-0.09] | 0.05 [0.04-0.07] |
|  | Malawi | 0.07 [0.04-0.10] | 0.19 [0.16-0.22] | 0.04 [0.02-0.05] | 0.03 [0.01-0.04] |
|  | Pakistan | -0.02 [-0.05-0.01] | -0.05 [-0.08--0.02] | 0.00 [-0.02-0.02] | 0.03 [0.01-0.05] |
|  | Peru | 0.00 [-0.04-0.04] | -0.04 [-0.07--0.01] | 0.01 [-0.02-0.04] | -0.02 [-0.04-0.01] |
|  | The Gambia | 0.11 [0.08-0.14] | -0.03 [-0.04--0.01] | -0.14 [-0.16--0.12] | 0.01 [0.00-0.03] |
|  | Overall | 0.06 [-0.04-0.23] | -0.04 [-0.24-0.2] | 0.01 [-0.15-0.12] | 0.00 [-0.13-0.06] |

Table G. Model Performance in the prediction of watery bacterial diarrhea by qPCR

| **Including biomarkers, and clinical and socio-demographic predictors** | | | | | |
| --- | --- | --- | --- | --- | --- |
| **Site** | **Sensitivity [95% CI]** | **Specificity [95% CI]** | **PPV [95% CI]** | **NPV [95% CI]** | **AUC [95% CI]** |
| Bangladesh | 0.84 [0.24-0.9] | 0.30 [0.24-0.87] | 0.26 [0.23-0.37] | 0.86 [0.79-0.9] | 0.58 [0.55-0.61] |
| Kenya | 0.78 [0.52-0.88] | 0.41 [0.30-0.60] | 0.13 [0.11-0.15] | 0.94 [0.91-0.96] | 0.56 [0.51-0.61] |
| Malawi | 0.51 [0.17-0.83] | 0.61 [0.08-0.91] | 0.12 [0.07-0.17] | 0.92 [0.79-0.94] | 0.53 [0.49-0.59] |
| Pakistan | 0.62 [0.15-0.77] | 0.46 [0.14-0.75] | 0.21 [0.11-0.26] | 0.82 [0.68-0.87] | 0.51 [0.46-0.56] |
| Peru | 0.56 [0.30-1.00] | 0.75 [0.22-0.90] | 0.07 [0.02-0.13] | 0.98 [0.93-1.00] | 0.58 [0.49-0.72] |
| The Gambia | 0.45 [0.15-0.81] | 0.56 [0.16-0.85] | 0.27 [0.20-0.32] | 0.79 [0.71-0.83] | 0.52 [0.49-0.56] |
| Overall | 0.55 [0.52-0.76] | 0.51 [0.40-0.66] | 0.16 [0.08-0.27] | 0.88 [0.79-0.97] | 0.55 [0.51-0.59] |
| **Excluding biomarkers** | | | | | |
| **Site** | **Sensitivity [95% CI]** | **Specificity [95% CI]** | **PPV [95% CI]** | **NPV [95% CI]** | **AUC [95% CI]** |
| Bangladesh | 0.52 [0.05-0.84] | 0.54 [0.12-0.91] | 0.24 [0.14-0.28] | 0.79 [0.72-0.83] | 0.51 [0.49-0.54] |
| Kenya | 0.55 [0.18-0.89] | 0.34 [0.03-0.84] | 0.09 [0.07-0.17] | 0.86 [0.62-0.92] | 0.53 [0.49-0.58] |
| Malawi | 0.38 [0.18-0.61] | 0.43 [0.22-0.64] | 0.07 [0.04-0.09] | 0.87 [0.83-0.90] | 0.56 [0.51-0.62] |
| Pakistan | 0.56 [0.08-0.96] | 0.52 [0.12-0.80] | 0.19 [0.08-0.25] | 0.81 [0.73-0.93] | 0.52 [0.47-0.57] |
| Peru | 0.8 [0.00-1.00] | 0.46 [0.39-0.78] | 0.05 [0.00-0.08] | 0.99 [0.95-1.00] | 0.54 [0.46-0.66] |
| The Gambia | 0.32 [0.10-0.89] | 0.58 [0.18-0.8] | 0.19 [0.12-0.28] | 0.75 [0.72-0.85] | 0.51 [0.49-0.54] |
| Overall | 0.51 [0.39-0.71] | 0.49 [0.37-0.57] | 0.14 [0.05-0.23] | 0.82 [0.77-0.97] | 0.52 [0.51-0.56] |

PPV- Positive Predictive value; NPV-Negative Predictive value; AUC- Area under the Curve

Table H. Comparison of bootstrapped AUCs for watery bacterial diarrhea prediction with iterative biomarker inclusion

| **Validation site** | **Incremental mean difference in AUC (95% CI)** | | | |
| --- | --- | --- | --- | --- |
|  | **Clinical predictors + HAEM vs. Clinical predictors** | **Clinical predictors + HAEM**  **and MPO vs. Clinical predictors + HAEM** | **Clinical predictors + HAEM, MPO, CAL vs. Clinical predictors + HAEM + MPO** | **Clinical predictors + HAEM, MPO, CAL, NGAL vs. Clinical predictors + HAEM + MPO + CAL** |
| Bangladesh | 0.01 [-0.03-0.03] | 0.03 [0.01-0.07] | 0.00 [-0.02-0.02] | 0.03 [0.01-0.05] |
| Kenya | 0.00 [-0.07-0.08] | 0.02 [-0.02-0.07] | 0.00 [-0.03-0.03] | 0.02 [-0.01-0.05] |
| Malawi | 0.00 [-0.06-0.07] | 0.00 [-0.06-0.04] | 0.01 [-0.08-0.06] | -0.01 [-0.04-0.06] |
| Pakistan | 0.03 [-0.02-0.1] | -0.02 [-0.08-0.03] | 0.00 [-0.05-0.05] | 0.01 [-0.05-0.05] |
| Peru | -0.01 [-0.11-0.09] | -0.02 [-0.15-0.11] | 0.04 [-0.09-0.17] | 0.04 [-0.05-0.18] |
| The Gambia | 0.00 [-0.06-0.04] | -0.01 [-0.06-0.05] | 0.01 [-0.03-0.04] | 0.01 [-0.03-0.04] |
| Overall | 0.00 [-0.07-0.08] | 0.00 [-0.10-0.07] | 0.01 [-0.07-0.11] | 0.02 [-0.04-0.10] |

Table I. Comparison of bootstrapped sensitivities and specificities for watery bacterial diarrhea prediction with iterative biomarker inclusion

| **Metric** | **Validation site** | **Incremental mean difference in AUC (95% CI)** | | | |
| --- | --- | --- | --- | --- | --- |
|  |  | **Clinical predictors + HAEM vs. Clinical predictors** | **Clinical predictors + HAEM**  **and MPO vs. Clinical predictors + HAEM** | **Clinical predictors + HAEM, MPO, CAL vs. Clinical predictors + HAEM + MPO** | **Clinical predictors + HAEM, MPO, CAL, NGAL vs. Clinical predictors + HAEM + MPO + CAL** |
| Sensitivity | Bangladesh | 0.19 [0.15-0.24] | -0.15 [-0.2--0.1] | 0.13 [0.09-0.16] | 0.06 [0.03-0.10] |
|  | Kenya | 0.32 [0.23-0.4] | -0.15 [-0.22--0.08] | 0.06 [0.00-0.12] | -0.05 [-0.10-0.00] |
|  | Malawi | 0.30 [0.21-0.40] | -0.46 [-0.56--0.36] | 0.30 [0.21-0.39] | -0.07 [-0.12--0.03] |
|  | Pakistan | -0.06 [-0.12-0.00] | 0.26 [0.19-0.33] | -0.27 [-0.34--0.2] | -0.29 [-0.37--0.22] |
|  | Peru | 0.15 [0.00-0.33] | -0.54 [-0.8--0.29] | 0.46 [0.25-0.67] | 0.15 [0.00-0.33] |
|  | The Gambia | -0.14 [-0.19--0.09] | -0.05 [-0.09--0.02] | 0.68 [0.63-0.73] | -0.47 [-0.52--0.41] |
|  | Overall | -0.14 [-0.19--0.09] | -0.05 [-0.09--0.02] | 0.68 [0.63-0.73] | -0.47 [-0.52--0.41] |
| Specificity | Bangladesh | -0.16 [-0.18--0.14] | 0.19 [0.16-0.21] | -0.13 [-0.16--0.11] | -0.04 [-0.06--0.02] |
|  | Kenya | -0.16 [-0.19--0.13] | 0.17 [0.15-0.2] | -0.05 [-0.08--0.03] | 0.09 [0.07-0.12] |
|  | Malawi | -0.3 [-0.33--0.27] | 0.61 [0.58-0.64] | -0.23 [-0.26--0.20] | 0.04 [0.02-0.07] |
|  | Pakistan | -0.13 [-0.17--0.1] | -0.16 [-0.2--0.13] | 0.39 [0.35-0.43] | 0.15 [0.12-0.18] |
|  | Peru | -0.1 [-0.14--0.06] | 0.63 [0.59-0.67] | -0.12 [-0.14--0.09] | -0.04 [-0.06--0.01] |
|  | The Gambia | 0.13 [0.10-0.15] | 0.12 [0.10-0.14] | -0.58 [-0.61--0.55] | 0.47 [0.44-0.50] |
|  | Overall | -0.12 [-0.31-0.14] | 0.26 [-0.18-0.65] | -0.12 [-0.60-0.41] | 0.11 [-0.05-0.49] |
