# Supplementary material for: Performance of fecal inflammatory biomarkers to identify watery shigellosis: Findings from the Enterics for Global Health (EFGH) Shigella surveillance study
Source: PLoS Negl Trop Dis. 2026 Jun 1;20(6):e0014025. doi: 10.1371/journal.pntd.0014025 (PMC13235866; doi:10.1371/journal.pntd.0014025)
Supplement: Attachment — Submitted filename: Point_by_Point Response to Reviewer Comments_01.04.2026.docx [file pntd.0014025.s003.docx]

**Point-by-point response to Editor/Reviewer Comments**

We sincerely appreciate the invaluable comments and suggestions provided by the editor and the reviewers, which we believe have contributed in improving the quality of this work. Below, we present a point-by-point response to each of the remarks (in italic), clearly highlighting the revisions made in the manuscript (with line numbers (**highlighted in bold**) referenced in the marked version) and offering clarifications where needed.

**Editorial Comments to Author:**

**Journal Requirements:**

**Comment 1:** Please ensure that the CRediT author contributions listed for every co-author are completed accurately and in full.
At this stage, the following Authors/Authors require contributions: Sara Kim. Please ensure that the full contributions of each author are acknowledged in the "Add/Edit/Remove Authors" section of our submission form. The list of CRediT author contributions may be found here: <https://journals.plos.org/plosntds/s/authorship#loc-author-contributions>

**Response 1:** Thank you for this comment. We have reviewed author contributions for all authors and have updated contributions for Sara Kim.

**Comment 2**: Please amend your detailed Financial Disclosure statement. This is published with the article. It must therefore be completed in full sentences and contain the exact wording you wish to be published.
State the initials, alongside each funding source, of each author to receive each grant. For example: "This work was supported by the National Institutes of Health (####### to AM; ###### to CJ) and the National Science Foundation (###### to AM).".
If you did not receive any funding for this study, please simply state: The authors received no specific funding for this work.
**Response 2:** Thank you for this comment. We have revised the financial disclosure statement to clearly include the funding source and the initials of each relevant author.

**Comment 3**: **Figure resubmission:** While revising your submission, we strongly recommend that you use PLOS’s NAAS tool (<https://ngplosjournals.pagemajik.ai/artanalysis>) to test your figure files. NAAS can convert your figure files to the TIFF file type and meet basic requirements (such as print size, resolution), or provide you with a report on issues that do not meet our requirements and that NAAS cannot fix.

After uploading your figures to PLOS’s NAAS tool - <https://ngplosjournals.pagemajik.ai/artanalysis>, NAAS will process the files provided and display the results in the "Uploaded Files" section of the page as the processing is complete. If the uploaded figures meet our requirements (or NAAS is able to fix the files to meet our requirements), the figure will be marked as "fixed" above. If NAAS is unable to fix the files, a red "failed" label will appear above. When NAAS has confirmed that the figure files meet our requirements, please download the file via the download option, and include these NAAS processed figure files when submitting your revised manuscript.

**Response 3:** We appreciate this comment. We have regenerated the figures using NAAS and confirm that all the figures meet the required specifications.

**Comment 4**: **Reproducibility:** To enhance the reproducibility of your results, we recommend that authors of applicable studies deposit laboratory protocols in [protocols.io](http://protocols.io), where a protocol can be assigned its own identifier (DOI) such that it can be cited independently in the future. Additionally, PLOS ONE offers an option to publish peer-reviewed clinical study protocols. Read more information on sharing protocols at <https://plos.org/protocols?utm_medium=editorial-email&utm_source=authorletters&utm_campaign=protocols>

**Response 4:** The study protocol for both the primary EFGH Study and the Biomarker Sub-study were made publicly available. (<https://academic.oup.com/ofid/issue/11/Supplement_1>). This has been updated accordingly under the data availability statement in Lines **515-519**.

**Reviewer Comments to Author:

Reviewer 1:**

**Comment 1:** Reviewer #1: Bacterial diarrhea, particularly that caused by Shigella, is typically an invasive infection characterized by the presence of blood in the stool. However, in some cases, patients with invasive bacterial diarrhea may not have visibly bloody stools. This study aims to identify differences among patients with diarrhea (without visible blood in the stool) in order to better inform clinical practice. I believe this point should be emphasized in the background. Alternatively, in line 55, the phrase “watery diarrhea” could be revised to “watery diarrhea (non-bloody)” to clarify the study objective more clearly.

**Response 1:** Thank you for this helpful comment. We have clarified that watery diarrhea refers to non-bloody diarrhea to better align with the study objective. The abstract and background have been revised accordingly (Lines **55, 121, 149,** and **156**) to improve clarity.

**Comment 2:** In the predictors section: The authors could define dehydration status at line 235 (i.e., severe dehydration and some dehydration, mentioned at line 325).

**Response 2:** Thank you for this comment. We have clarified that this is the WHO dehydration assessment , which classifies patients into three categories: no dehydration, some dehydration, and severe dehydration under the predictors in the Methods section in Lines **239-240.**

**Comment 3:** The authors use the variable “Site-specific indicator of the Shigella transmission season (defined as the 3 months with the highest Shigella prevalence at each site)” as a predictor. The authors may explain the rationale for selecting this variable. Is diarrhea caused by Shigella a disease with clear seasonal variation? Additionally, is there any difference in the timing of Shigella diarrhea cases? For example, are there differences in the months with the highest number of Shigella diarrhea cases among the six cities included in the study? Furthermore, does the peak period of Shigella diarrhea vary from year to year? If so, it may be difficult to use this variable as a reliable predictor of the causative pathogen.

**Response 3:** Thank you for this comment. We included the site-specific Shigella seasonality indicator based on well-established evidence that Shigella transmission exhibits seasonal patterns, often aligning with rainfall and environmental conditions that facilitate transmission. This makes seasonality a potentially informative contextual predictor. To construct this variable, we aggregated calendar months across the full study period and defined, for each site, the three months with the highest observed Shigella prevalence. We observed a partial alignment in mid-year seasonality (May–August) in Bangladesh, Kenya, Peru, and The Gambia, while Malawi and Pakistan showed more heterogeneous or multi-peaked patterns, indicating site-level differences in transmission dynamics. This supports the rationale for using a site-specific, rather than global, seasonal indicator. We also conducted a sensitivity analysis excluding the seasonality variable, which resulted in only a modest reduction in performance (AUC decreased by ~3% to 0.72), suggesting that the model does not rely heavily on this predictor. Furthermore, the final simplified clinical score we propose does not include the seasonality variable; it uses only age, dichotomized hemoglobin concentration, and stool frequency, while maintaining comparable performance to the full model (AUC = 0.75).

**Comment 4:** In this study, 55.8% of participants were male; therefore, I think the use of the word “most” in line 323 may not be appropriate. It may be more accurate to state that “more than half were male.”

**Response 4:** The statement has been updated accordingly in Line 325, Thank you.

**Comment 5:**In Table 1: The authors may consider adding p-values to compare the characteristics across the six cities.

**Response 5:** Thank you for this comment. We have updated Table to include p-values with a footnote highlighting how p-values were estimated. We have also added a sentence summarizing site-level heterogeneity observed across all variables except for child sex in Lines **333-334.**

**Comment 6:** Regarding age groups, the authors may also explain the rationale for categorizing age as 6–11, 12–17, 18–23, and 24–35 months, rather than 6–11, 12–17, and 18–35 months as presented in Table 4.

**Response 6:** Thank you for this comment to provide the rationale behind the age categorizations in Table 1. The use of these different age categorizations reflects two complementary objectives in the analysis. The finer 6-month stratification (6–11, 12–17, 18–23, and 24–35 months) presented in Table 1 was used to characterize the epidemiology of Shigella in greater detail in line with the significant changes in host immunity, nutritional status, and dietary exposures that occur during these early developmental windows. This approach aligns with known age-related differences in risk and allows for more granular assessment of how Shigella prevalence varies across early childhood. Conversely, the broader age categories (6–11, 12–17, and 18–35 months) used in the clinical score presented in Table 4 were selected to optimize model performance and usability. Combining the older age groups improved model stability and parsimony without materially compromising predictive accuracy, while also making the final tool more practical for clinical application. In conclusion, the differing categorizations reflect a balance between epidemiological resolution and predictive efficiency and usability in the final model.

**Reviewer 2:**

**Comment 1:** No new analyses are required for acceptance. The objectives of the study are clearly stated; the study design, sample size, and statistics are appropriate; and there are no ethical or regulatory concerns.

**Response 1:** Thank you for this comment

**Comment 2:** The results presented match the analysis plan and the results are clearly presented. However, the figures had low resolution, but that could be due to the PDF generated from the files. I also did not see any figure legends included in the manuscript text or attached with the figures (for figures 1-3 in the manuscript).

**Response 2:** Thank you for this comment. The figures were generated using NAAS, as recommended by the journal, and we confirm that all figures meet the required specifications. Figure legends for the main figures have been incorporated in-text (Lines **349** [Fig 1], **365** [Fig 2], and **368** [Fig 3]). Legends for the supplementary figures are provided at the end of the manuscript, following the references (Lines **690-714**).

**Comment 3:** The manuscript highlights analysis of fecal inflammatory biomarkers to identify watery shigellosis. Fecal samples were collected across six established study sites and analyzed in children aged 6-35 months presenting with watery diarrhea. Fecal samples were analyzed by culturing or qPCR, and for the presence of several biomarkers of inflammatory diarrhea. A model was then generated to predict watery diarrhea as a result of Shigella infection. Several iterations of the model were tested, but the full model incorporating all predictors of watery diarrhea had the best results. The authors conclude that one biomarker in particular, fecal hemoglobin, improved the prediction scores. Consideration of fecal hemoglobin could improve diagnostics and ultimately treatment for shigellosis.

Overall, the authors present a thorough study with important reference standards for the data; describe the methodology, outcomes, and model generation well; and address limitations. Overall, the model performs well, especially when considering that the authors aim to detect Shigella based on watery diarrhea and not when dysentery (inflammatory diarrhea) is present. Here is where a significant strength of the study is noted, since the impact of Shigella infections are often limited by variability in symptoms and difficult in detecting the pathogen by culture or qPCR. Thus, this model may help with clinical diagnosis and ultimately treatment decisions. The manuscript is presented well, and no major weaknesses were identified. However, a few minor text edits are suggested for clarifications or to further enhance the manuscript, especially more general readers:

**Comment 4:** Is antibiotic treatment restricted with watery diarrhea since it can resolve with supportive care? Or is the issue of limitation due to unknown etiology and/or concerns about AMR?

**Response 4:** Thank you for this comment. The restriction on antibiotic use for watery diarrhea reflects a combination of clinical and public health considerations. In the absence of diagnostic testing, dysentery has a higher likelihood of *Shigella* compared to watery diarrhea hence the risk-benefit ratio for empirical treatment is unfavorable. Additionally, the growing concerns on AMR necessitates restricting antibiotics to higher-probability bacterial syndromes (dysentery suggestive of *Shigella*) to help preserve their effectiveness. This clarification has been added in Lines **116-119.**

**Comment 5:** 2. For studies like this one presented, what is considered a good or excellent AUC? The authors discuss other studies and compare their results well, but a statement of a good to excellent AUC range would be helpful.

**Response 5:** Generally, an AUC of 0.5 indicates no discriminatory ability (the test cannot distinguish between those with and without the condition), 0.7–0.8 is considered acceptable, 0.8–0.9 is excellent, and above 0.9 is outstanding. This has been updated in the discussion in Lines **449-451** and supporting reference provided.

**Comment 6:** Could the calculated AUC have been lowered due to false negatives with rectal swab culturing or qPCR?
**Response 6:** Thank you for this comment. While qPCR and rectal swabs may yield some false negatives, potentially lowering the observed AUC, comparisons in the parent study showed similar Shigella detection between whole stools and rectal swabs. Additionally, we used Shigella-attributable diarrhea based on quantitative qPCR loads, which helps distinguish true infection from colonization. The overall trends, especially the predictive contribution of fecal hemoglobin, remain robust across sites and sample types.

**Comment 7:**. Since Shigella diagnosis can be difficult for multiple reasons, this approach also helpful for surveillance and more thoroughly understanding the global impact of Shigella.

**Response 7:** Thank you for this comment. We agree our biomarker-based approach offers a practical, low-cost strategy to enhance clinical decision-making and surveillance by improving detection of watery Shigella cases, guiding more targeted antibiotic use, and providing a fuller picture of disease burden beyond the dysentery definition, even if it does not fully match qPCR performance.
